# Adult T-Cell Leukemia/Lymphoma-Related Ocular Manifestations: Analysis of the First Large-Scale Nationwide Survey

**DOI:** 10.3389/fmicb.2018.03240

**Published:** 2019-01-08

**Authors:** Koju Kamoi, Akihiko Okayama, Shuji Izumo, Isao Hamaguchi, Kaoru Uchimaru, Arinobu Tojo, Kyoko Ohno-Matsui

**Affiliations:** ^1^Department of Ophthalmology and Visual Science, Graduate School of Medical and Dental Sciences, Tokyo Medical and Dental University, Tokyo, Japan; ^2^Department of Hematology/Oncology, Research Hospital, The Institute of Medical Science, The University of Tokyo, Tokyo, Japan; ^3^Department of Rheumatology, Infectious Diseases and Laboratory Medicine, Faculty of Medicine, University of Miyazaki, Miyazaki, Japan; ^4^Division of Molecular Pathology, Center for Chronic Viral Diseases, Graduate School of Medical and Dental Sciences, Kagoshima University, Kagoshima, Japan; ^5^Department of Safety Research on Blood and Biological Products, National Institute of Infectious Diseases, Tokyo, Japan; ^6^Laboratory of Tumor Cell Biology, Department of Medical Genome Sciences, Graduate School of Frontier Sciences, The University of Tokyo, Tokyo, Japan

**Keywords:** adult T-cell leukemia, ocular manifestations, nationwide survey, intraocular infiltration, human T-cell leukemia virus type 1

## Abstract

Adult T-cell leukemia/lymphoma (ATL) is a rare and aggressive T-cell malignancy with a high mortality rate, resulting in a lack of information among ophthalmologists. Here, we investigated the state of ophthalmic medical care for ATL and ATL-related ocular manifestations by conducting the first large-scale nationwide survey in Japan. A total of 115 facilities were surveyed, including all university hospitals in Japan that were members of the Japanese Ophthalmological Society and regional core facilities that were members of the Japanese Ocular Inflammation Society. The collected nationwide data on the state of medical care for ATL-related ocular manifestations and ATL-associated ocular findings were categorized, tallied, and analyzed. Of the 115 facilities, 69 (60%) responded. Overall, 28 facilities (43.0%) had experience in providing ophthalmic care to ATL patients. ATL-related ocular manifestations were most commonly diagnosed “based on blood tests and characteristic ophthalmic findings.” By analyzing the 48 reported cases of ATL-related ocular manifestations, common ATL-related ocular lesions were intraocular infiltration (22 cases, 45.8%) and opportunistic infections (19 cases, 39.6%). All cases of opportunistic infection were cytomegalovirus retinitis. Dry eye (3 cases, 6.3%), scleritis (2 cases, 4.2%), uveitis (1 case, 2.1%), and anemic retinopathy (1 case, 2.1%) were also seen. In conclusion, intraocular infiltration and cytomegalovirus retinitis are common among ATL patients, and ophthalmologists should keep these findings in mind in their practice.

## Introduction

Human T-cell leukemia virus type 1 (HTLV-1) was the first retrovirus found to infect and cause disease in humans (Hinuma et al., 1981). The routes of transmission are primarily through sexual contact to adults, and through breast milk to infants (Iwanaga et al., 2009). Such infections are prevalent in Melanesia, the Caribbean Islands, Central and South America, and Central Africa, as well as areas such as Kyushu and Okinawa in Southwestern Japan (Watanabe, 2011). Among developed nations, Japan is estimated to have the highest proportion of infected individuals, with approximately 1 million (Satake et al., 2012) out of a total population of 126 million.

HTLV-1 causes diseases such as adult T-cell leukemia/lymphoma (ATL) (Yoshida et al., 1984), HTLV-1-associated myelopathy (HAM) (Osame et al., 1986), and HTLV-1 uveitis (HU) (Mochizuki et al., 1992a,b; Kamoi and Mochizuki, 2012b). Ophthalmic care is required for HU and ATL-related ocular manifestations (Kamoi and Mochizuki, 2012a), but ATL-related ophthalmic manifestations remain relatively obscure among ophthalmologists (Kamoi and Mochizuki, 2012a).

ATL is a rare disease, and the annual rate of ATL developing among HTLV-1 carriers is estimated to be between 7.7 and 8.7 per 10,000 people (Satake et al., 2015). As for prognosis, median overall survival time was 7.7 months according to the simplified ATL prognostic index report (Katsuya et al., 2012). Subsequent central nervous system invasion of ATL occurs in 10–20% of cases (Bazarbachi et al., 2011). In treatment, many therapeutic agents have been used to improve this poor prognosis. Representative available therapies include intensive multi-agent chemotherapy (Tsukasaki et al., 2012), interferon-a combined with zidovudine (Gill et al., 1995; Hermine et al., 1995), and an anti-CCR4 antibody (mogamulizumab) (Ishida et al., 2012). Hematopoietic stem cell transplantation has recently been reported to achieve long-lasting remission (Zell et al., 2016) and the effectiveness of Tax peptide-pulsed dendritic cell vaccine has been reported (Suehiro et al., 2015).

As a result of its rarity and poor prognosis, very few reports have described ATL-related ocular manifestations (Kohno et al., 1993; Shibata et al., 1997; Kamoi et al., 2016; Hirano et al., 2017), and the details of ocular lesions remain unclear. Given this background of limited information, we conducted a large-scale survey on the state of ophthalmic practice for ATL patients in Japan, where there are a large number of patients with ATL caused by HTLV-1 infection, to analyze and assess ATL-related ocular manifestations.

## Materials and Methods

All study protocols for this investigation were approved by the ethics review committees of the Tokyo Medical and Dental University and the Institute of Medical Science at the University of Tokyo, in accordance with the tenets of the Declaration of Helsinki. In March 2015, a questionnaire survey regarding the state of ophthalmic medical care for ATL and ATL-related ocular manifestations was conducted on a total of 115 facilities, including all university hospitals throughout Japan that were members of the Japanese Ophthalmological Society and all ophthalmic facilities providing medical care for ocular inflammation that were members of the Japanese Ocular Inflammation Society.

**FIGURE 1:**
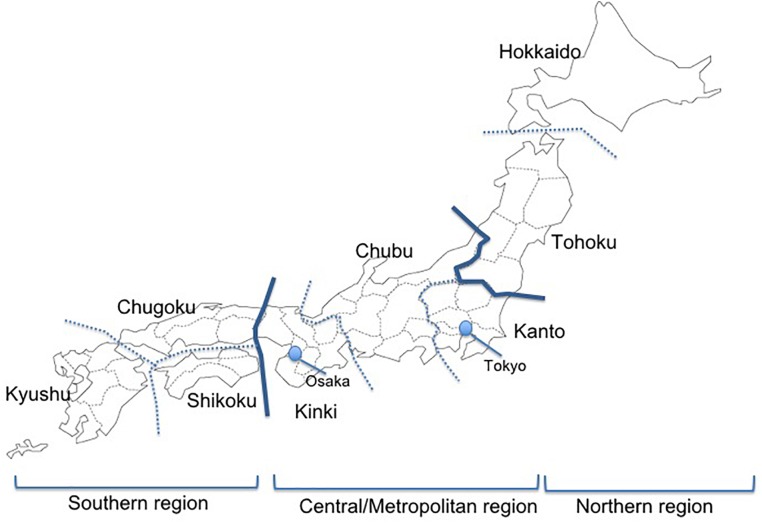
A map showing the regions of Japan. The Kanto and Kinki regions are metropolitan areas in Japan with population growth in recent years due to population movements, and the Kyushu region has a high prevalence of HTLV-1.

Questions focused on facility locations, classification, experience with ophthalmic care for ATL patients, methods for diagnosing ocular manifestations of ATL, ocular findings observed with ATL-related ocular manifestations, and frequency of ATL-related ocular manifestations (Figure 1 and Table 1). ATL-related ocular manifestations were defined as ocular disorders attributed to ATL. We suggested several expected ATL-related ocular manifestations in consideration of previous reports, and added a blank field to allow respondents to report other manifestations. Considering the rarity of the pathology, relatively few patients with ATL-related ocular lesions were expected to be registered by each facility, so we asked each facility to report all patients registered as showing ATL-related ocular manifestations as of March 2015.

**Table 1:** Questionnaire used for assessing the state of medical care for ATL-related ocular manifestations and ATL-associated ocular findings.

Questionnaire	Answer
(1) Has your department ever provided ophthalmic care for ATL patients?	□Yes ( ) cases □No
(2) How do you diagnose ATL-related ocular manifestations?	□Based on a blood test (positive for HTLV-1 antibodies) and the characteristic ophthalmic findings □Based on a test of intraocular fluid (PCR test) and the characteristic ophthalmic findings □Other (Please provide the specifics: )
(3) Do you exclude other forms of uveitis when diagnosing an ATL- related ocular manifestation?	□Yes □No □Other (Please provide the specifics: )
(4) What are the findings that have been observed in ATL-related ocular manifestations?	□Intraocular infiltration ( ) cases □Opportunistic infection ( ) cases (Please provide the specifics: ) □Dry eye ( ) cases □Other ( ) cases (Please provide the specifics: )
(5) Do you think the number of ATL-related ocular manifestations is on the rise in recent years?	□On the rise □On the decline □Unchanged


Only valid responses to questions were included in the statistical analyses. If a respondent left a question blank, only that blank response was excluded from analysis. Data on categories pertaining to medical care for ATL-related ocular manifestations were tallied and analyzed.

## Results

Of the 115 facilities, 69 (60.0%) responded. Responses were received from facilities throughout Japan, with those in the Kanto region accounting for 39.1% of overall respondents and those in the high-prevalence Kyushu region accounting for 13.0% (Table 2). By type of facility, 55.0% of overall respondents were advanced treatment hospitals, 42.0% were hospitals, and 2.9% were clinics.

**Table 2:** Number of facilities responding to the questionnaire.

Northern	Central/Metropol Itan			Southern		Total
Hokkaido/Tohoku	Kanto	Chubu	Kinki	Chugoku/Shikoku	Kyushu	
5 (7.2%)	48 (69.5%)			16 (23.2%)		69
5 (7.2%)	27 (39.1%)	9 (13.0%)	12 (17.4%)	7 (10.1%)	9 (13.0%)	


In terms of experience in providing ophthalmic care to ATL patients, 43.0% of facilities indicated that they have such experience throughout Japan (Table 3A). In particular, 88.9% of facilities in the Kyushu region of Southern Japan have experience with ophthalmic treatment for ATL patients, as do 80.0% of facilities in the Hokkaido/Tohoku region of Northern Japan. Looking at central/metropolitan areas, 36.4% of facilities in the Kinki region, 26.9% in the Kanto region, and 14.2% in the Chubu region have such experience.

**Table 3A:** Experience with medical care for ATL patients.

	Northern (*n* = 5)	Central/Metropolitan (n = 44)			Southern (*n* = 16)		
Experience of medical care for ATL patients	Hokkaido/Tohoku (*n* = 5)	Kanto (*n* = 26)	Chubu (*n* = 7)	Kinki (*n* = 11)	Chugoku/Shikoku (*n* = 7)	Kyushu (*n* = 9)	Total
Yes	80.0%	29.7%			75.0%		43.0%
	80.0%	26.9%	14.2%	36.4%	57.1%	88.9%	
No/Un-identified	20.0%	70.3%			25.0%		57.0%
	20.0%	73.0%	85.7%	63.6%	42.9%	11.1%	


The survey showed that ATL-related ocular manifestations are most commonly diagnosed “based on blood tests and characteristic ophthalmic findings” (65.0% of facilities), followed by “based on consulting hematologists and ophthalmic examination,” which was a specific option provided in the questionnaire (18.3% of facilities), and “based on tests of intraocular fluid and characteristic ophthalmic findings” (16.7% of facilities) (Table 3B). Regarding the question of whether differential diagnosis is practiced by excluding other forms of uveitis when diagnosing ATL-related ocular manifestations, 90.6% responded “Yes,” and the remaining 9.4% commented “No experience.”

**Table 3B:** Diagnostic methods for ATL ocular manifestations.

Diagnostic Methods	Northern (*n* = 8)	Central/Metropolitan (*n* = 36)			Southern (*n* = 16)		
	Hokkaido/Tohoku (*n* = 8)	Kanto (*n* = 20)	Chubu (*n* = 5)	Kinki (*n* = 11)	Chugoku/Shikoku (n = 6)	Kyushu (*n* = 10)	Total
Blood test and ophthalmic examination	50.0%	69.4%			62.5%		65.0%
	50.0%	70.0%	80.0%	63.6%	83.3%	50.0%	
Intraocular fluid test and ophthalmic examination	37.5%	11.1%			18.8%		16.7%
	37.5%	10.0 %	0.0%	18.2%	0.0%	30.0%	
Consulting Hematologists and ophtalmic examination	12.5%	19.4%			18.8%		18.3%
	12.5%	20.0%	20.0%	18.2 %	16.7%	20.0%	


Respondents reported 48 cases of ATL-related ocular manifestations. By region, the number of cases reported was highest in the Kanto region (22 cases), followed by the Kinki and Chugoku regions (Table 4). The most common type of ATL-related ocular manifestation was intraocular infiltration (22 patients, 45.8%), followed by opportunistic infection (19 patients, 39.6%) and dry eye (3 patients, 6.35%), with scleritis indicated in the blank field (2 patients, 4.2%). Additional responses included a case of uveitis that resolved after steroid treatment (2.1%) and a case of anemic retinopathy (2.1%), which is commonly seen with leukemia. All cases of opportunistic infection involved cytomegalovirus retinitis (CMVR). In addition to CMVR, one case of superinfection with *Toxoplasma* and two cases of herpesvirus were also reported (Table 4).

**Table 4:** ATL-related ocular manifestations.

Manifestations	Number of patients							
	Northern	Central/Metropolitan			Southern		
	Hokkaido/Tohoku	Kanto	Chubu	Kinki	Chugoku/Shikoku	Kyushu	Total
Intraocular infiltration	2	13			7		22 (45.8%)
	2	4	1	8	5	2	
Opportunistic infection	1	15			3		19 (39.6%)
(Cytomegalovirus ^∗^)	(1)	(15)	(0)	(0)	(1)	(2)	(19)
(Herpesvirus^∗∗^)	(0)	(0)	(0)	(0)	(0)	(2)	(2)
(Toxoplasma^∗∗^)	(0)	(0)	(0)	(0)	(0)	(1)	(1)
Dry eye	0	2			1		3 (6.3%)
	0	2	0	0	0	1	
Scleritis	0	0			2		2 (4.2%)
	0	0	0	0	2	0	
Uveitis	0	0			1		1 (2.1%)
	0	0	0	0	0	1	
Anemic retinopathy	0	1			0		1 (2.1%)
	0	1	0	0	0	0	


*^∗^All cases of opportunistic infection were cytomegalovirus retinitis. ^∗∗^In addition to cytomegalovirus retinitis, one case of superinfection with toxoplasma and two cases of herpesvirus were reported.*

Among the responding facilities, 87.2% indicated that no changes in the number of cases of ATL-related ocular manifestations had been seen in recent years. Increases were reported by 4.3% of facilities, all from the Kanto region. On the other hand, 8.5% of facilities (from the Kyushu, Chugoku, and Kanto regions) reported decreases.

## Discussion

With ATL representing an extremely rare form of leukemia with a high mortality rate (Katsuya et al., 2012), most cases of ATL-related ocular manifestation have been reported as ocular lesions in the format of a case report, resulting in an extreme lack of systemic information (Kamoi and Mochizuki, 2012a). Given the large number of HTLV-1-infected individuals in Japan, presumably representing the largest number of ATL patients among advanced nations, we were able to collect information on 48 patients, representing an unprecedentedly large number, in the first large-scale survey conducted in Japan. Our survey focused particularly on investigating which regions of Japan have treated a large number of cases of ocular lesions in ATL patients and what types of ocular lesions are associated with ATL.

With regard to distribution, ATL-related ocular manifestations have been treated throughout Japan (Tables 3A, 4), not just in regions with a high prevalence of HTLV-1. A large number of cases were seen in metropolitan areas such as the Kanto region (including Tokyo) and Kinki region (including Osaka). One possible interpretation is to attribute this finding to selection bias due to the nature of the questionnaire survey, as most ATL patients had been reported in southwestern Japan. However, the result might also reflect population movement-associated migration of HTLV-1-infected individuals to urban areas (Uchimaru et al., 2008).

With respect to diagnostic procedures, the survey revealed that a large number of facilities conduct blood tests that include testing for HTLV-1 antibodies to diagnose ATL, then render a diagnosis based on the characteristic ocular lesion. Approximately 20% of facilities conduct polymerase chain reaction (PCR) testing (Mochizuki et al., 2013) of intraocular fluid, representing a more precise method of diagnosis (Table 3A). This was attributed to the approval in Japan of PCR tests for intraocular fluid as advanced medical care in 2014.

As part of a measure to control such infections in Japan, pregnant women and blood donors have been screened for HTLV-1 antibodies in recent years (Iwanaga et al., 2009). Despite a decreasing trend in the number of infected individuals, 87.2% of responding facilities reported no changes in the number of cases of ATL-related ocular manifestation, and some responding facilities in the Kanto region have reported increases in the numbers of such cases. ATL develops after a long period of latency, with a high mean age of onset (around 60 years) (Iwanaga et al., 2012). Patients infected with HTLV-1 before the implementation of infection control measures have been beginning to develop the disease in recent years, so no reduction in such cases is expected in the near future.

**FIGURE 2:**
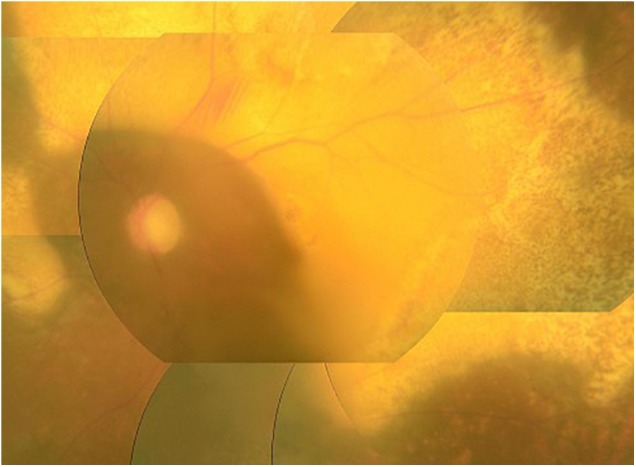
Typical clinical picture of ATL infiltration. Color fundus photograph showing yellowish-white infiltrative foci associated with protrusions in the retina.

**FIGURE 3:**
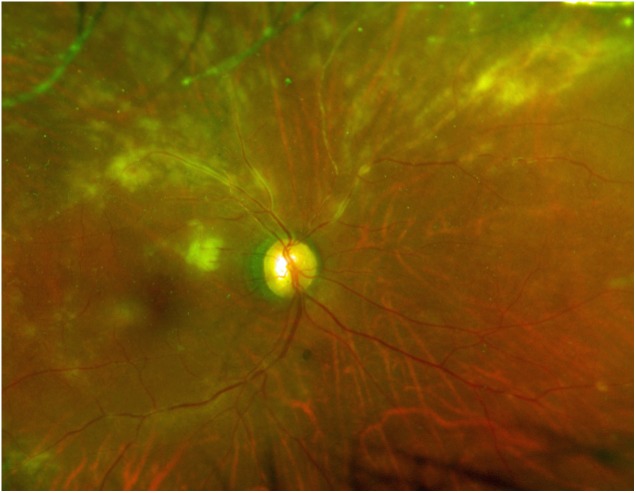
Typical clinical picture of cytomegalovirus retinitis. Color fungus photograph showing cytomegalic cell infiltration and widespread retinal disorganization.

As for ocular manifestations of ATL, our review of the literature on ocular lesions revealed case reports of ATL causing intraocular infiltration of ATL cells, with retinal hemorrhage/white patches, optic disk redness, and vitreous opacity as the major symptoms, as well as opportunistic infections associated with immunosuppression (Liu et al., 2010). The majority of the literature comprises case reports, and the types of ocular lesions that are common in ATL patients have thus remained unclear. The overwhelming majority of the 48 cases of ATL-related ocular lesions reported in this survey involved ocular infiltration (Figure 2) or opportunistic infection, with all cases of opportunistic infection being CMVR (Figure 3).

In large-scale surveys conducted in the past on acute myeloid leukemia, acute lymphatic leukemia, and chronic myeloid leukemia (Gordon et al., 2001; Russo et al., 2008; Khadka et al., 2014), the rate of ocular infiltration was not particularly high. In this survey, on the other hand, approximately half of the cases of ATL ocular lesions involved ocular infiltration. Compared with other forms of leukemia, ATL cells are thus much more adept at infiltrating the eyes. Recent studies have revealed that HTLV-1-infected T cells expressing C-C motif (CC) chemokine receptor 4 migrate to and infiltrate tissues such as the uvea, skin, parotid glands, and salivary glands, all of which express CC chemokine ligand (CCL) 17/CCL22 (Fukuda et al., 2003; Yoshie and Matsushima, 2015). This suggests that, through this mechanism, ATL cells migrate at a high rate to the uvea, resulting in ocular infiltration, and migrate to and infiltrate the lacrimal glands, causing dry eyes.

As for opportunistic infection, this study identified that all cases involved CMVR. CMV is a herpesvirus that infects 40–100% of adults. The clinical presentation of an active CMV infection often includes retinitis. CMV is well established as the most frequent pathogen of opportunistic infection in ATL patients (Suzumiya et al., 1993; Maeda et al., 2015) and CMV infection occurs more frequently in patients with ATL than in those with other leukemias (Funai et al., 1995). In ATL patients, HTLV-1-infected CD4-positive T cells can transform into malignant cells, losing the normal function of CD4-positive T cells. As a result, cellular immunity is severely impaired, resulting in frequent CMV infection.

The present results need to be considered in light of various limitations. This questionnaire survey asked questions regarding experience in providing medical care for ATL in major facilities throughout Japan. A more streamlined design may thus be needed to raise the response rate. Also, facilities without experience in treating ATL may well have been more likely to submit no response to the questionnaire. While the present results did not provide detailed information such as the main complaints, prognosis of visual acuity, sex ratio, or anatomical sites susceptible to infiltration, the results obtained provide valuable information regarding the medical care of such patients. We hope to work with each of the facilities in the future to further clarify the characteristics of ocular lesions in detail.

With the life prognosis of ATL patients improving in recent years due to improvements in treatment (Hermine et al., 2018; Marino-Merlo et al., 2018), ophthalmologists are increasingly likely to encounter opportunities to provide medical care to ATL patients. Ophthalmologists should keep in mind the high rates of intraocular infiltration and CMVR when examining patients with ATL.

## Author Contributions

KK designed the study and wrote the draft of the manuscript. AO designed the study. SI, IH, KU, AT, and KO-M contributed to analysis and interpretation of data, and assisted in the preparation of the manuscript. All authors critically reviewed and approved the final manuscript.

## Conflict of Interest Statement

The authorsdeclare that the research was conducted in the absence of any commercial or financial relationships that could be construed as a potential conflict of interest.
